# Human brain lesion-deficit inference remapped

**DOI:** 10.1093/brain/awu164

**Published:** 2014-06-28

**Authors:** Yee-Haur Mah, Masud Husain, Geraint Rees, Parashkev Nachev

**Affiliations:** 1 Institute of Neurology, UCL, London, WC1N 3BG, UK; 2 Department of Clinical Neurology, University of Oxford, Oxford OX3 9DU, UK; 3 Institute of Cognitive Neuroscience, UCL, London WC1N 3AR, UK; 4 Wellcome Trust Centre for Neuroimaging, UCL, London WC1N 3BG, UK

**Keywords:** lesion-deficit inference, focal brain injury, ischaemic brain injury

## Abstract

Our knowledge of the anatomical organization of the human brain in health and disease draws heavily on the study of patients with focal brain lesions. Historically the first method of mapping brain function, it is still potentially the most powerful, establishing the *necessity* of any putative neural substrate for a given function or deficit. Great inferential power, however, carries a crucial vulnerability: without stronger alternatives any *consistent* error cannot be easily detected. A hitherto unexamined source of such error is the structure of the high-dimensional distribution of *patterns* of focal damage, especially in ischaemic injury—the commonest aetiology in lesion-deficit studies—where the anatomy is naturally shaped by the architecture of the vascular tree. This distribution is so complex that analysis of lesion data sets of conventional size cannot illuminate its structure, leaving us in the dark about the presence or absence of such error. To examine this crucial question we assembled the largest known set of focal brain lesions (*n = *581), derived from unselected patients with acute ischaemic injury (mean age = 62.3 years, standard deviation = 17.8, male:female ratio = 0.547), visualized with diffusion-weighted magnetic resonance imaging, and processed with validated automated lesion segmentation routines. High-dimensional analysis of this data revealed a hidden bias within the multivariate patterns of damage that will consistently distort lesion-deficit maps, displacing inferred critical regions from their true locations, in a manner opaque to replication. Quantifying the size of this mislocalization demonstrates that past lesion-deficit relationships estimated with conventional inferential methodology are likely to be significantly displaced, by a magnitude dependent on the unknown underlying lesion-deficit relationship itself. Past studies therefore cannot be retrospectively corrected, except by new knowledge that would render them redundant. Positively, we show that novel machine learning techniques employing high-dimensional inference can nonetheless accurately converge on the true locus. We conclude that current inferences about human brain function and deficits based on lesion mapping must be re-evaluated with methodology that adequately captures the high-dimensional structure of lesion data.

## Abstract (precis)

Much of our knowledge of functional brain anatomy is based on lesion-deficit studies. Mah *et al.* show that the established methodology for conducting these — voxel-wise mass univariate inference — mislocalises function owing to complex correlations in natural patterns of damage across the brain; a problem soluble only by high-dimensional multivariate inference.

## Introduction

The study of patients with focal brain damage first revealed that the human brain has a functionally specialized architecture (Broca, 1861; Wernicke, 1874). Over the past century and a half such studies have been critical to identifying the distinctive neural substrates of language (Broca, 1861; Wernicke, 1874), memory (Scoville and Milner, 1957), emotion (Adolphs *et al.*, 1995; Calder *et al.*, 2000), perception (Goodale and Milner, 1992), decision-making (Bechara *et al.*, 1994), attention (Egly *et al.*, 1994; Mort *et al.*, 2003), and intelligence (Gläscher *et al.*, 2009), casting light on the anatomical basis of deficits resulting from dysfunction of the brain. Though functional imaging has revolutionized the field of brain function mapping in the last 20 years, the *necessity* of a brain region for a putative function—arguably the strongest test—can only be established by showing a deficit when the function of the region is disrupted. Inactivating brain areas experimentally cannot easily be done in humans; the special cases of transcranial magnetic and direct current stimulation, though potentially powerful, are restricted temporally to days and anatomically to accessible regions of cortex.

The only comprehensive means of establishing functional necessity thus remains the study of patients with naturally occurring focal brain lesions (Rorden and Karnath, 2004). Though single patients may sometimes be suggestive, robust, population-level inferences about lesion-deficit relationships require aggregation of data from many patients (Karnath *et al.*, 2004). Analogously to functional brain imaging, a statistical test comparing groups of patients with and without a deficit is iteratively applied point-by-point to brain lesion images parcellated into many volume units (voxels) (Bates *et al.*, 2003; Karnath *et al.*, 2004). Voxels that cross the significance threshold are then taken to identify the functionally critical brain areas whose damage leads to the deficit.

Crucially, this ‘mass-univariate’ approach assumes that the resultant structure-deficit localization is not distorted by co-incidental damage of other, non-critical loci in each patient: in other words, that damage to each voxel is independent of damage to any other. This cannot be assumed in the human brain. Collaterally damaged but functionally irrelevant voxels might be associated with voxels critical for a deficit through an idiosyncrasy of the pathological process—the distribution of the vascular tree, for example—while having no relation to the function of interest. Such associations would lead to a distortion of the inferred anatomical locus.

Importantly, these ‘parasitic’ voxel-voxel associations can be detected only by examining the multivariate pattern of damage across the entire brain, and across the entire group. Studying large numbers of patients with the standard approach simply exacerbates the problem, because such consistent error will also consistently displace inferred critical brain regions from their true locations. Equally, replicating a study with the same number of patients will replicate the error too: observing the same result across different research groups and epochs offers no reassurance.

Instead, the pattern of damage must be captured by a high-dimensional multivariate distribution that describes how the presence or absence of damage at every voxel within each brain image is related to damage to all other voxels. The presence of ‘parasitic’ voxel-voxel associations would then manifest as a hidden bias within the multivariate distribution, a complex correlation between individual patterns of damage apparent only in a high-dimensional space and opaque to inspection with simple univariate tools.

**Figure 1:**
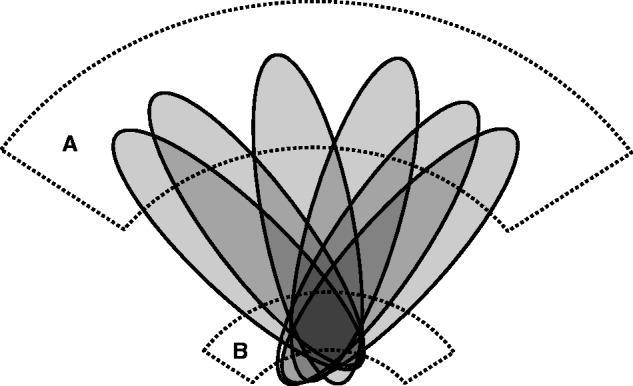
Illustration of how stereotyped patterns of brain damage (schematized in grey) across a set of patients can hypothetically mislocalize damage of any part of critical area A (in dotted lines) to the non-critical area B (in dotted lines). This will happen whenever the spatial variability of damage to a non-critical area is less for the group or factor of interest than for the critical area. Such stereotypy of damage—a hidden deep structure in the data—may occur where the lesions follow a consistent non-neural architecture, as is the case with vascular lesions.

To illustrate the problem, consider the 2D synthetic example in Fig. 1, where damage to any part of area ‘A’ alone may disrupt a putative function of interest, but ‘B’ plays no role in this function of interest. If the lesions used to map the functional dependence on A follow a stereotyped pattern where damage to any part of A is systematically associated with collateral damage to the non-critical area B, both areas might appear to be significantly associated even if B is irrelevant to the function of interest. Crucially, if the pattern of the lesions within each patient is such (for reasons to do with factors unconnected to function) that the spatial variability of damage to B is less than to A*,* B will not only be erroneously determined to be critical but will have a higher significance value for such an association than A*.* The apparent locus of a lesion-function deficit will therefore be displaced from A (the true locus) to B. Thus a hidden bias in the pattern of damage—hidden because it is apparent only when examining the pattern as a whole, in a multivariate way—distorts the spatial inference.

Whether or not such a hidden bias exists has not been previously investigated. Here we analyse the largest reported series of focal brain lesions (*n = *581) to show that it does exist, and that it compels a revision of previous lesion-deficit relationships within a wholly different inferential framework.

## Materials and methods

### Imaging data

Imaging data were collected from 581 patients attending University College London Hospitals for evaluation of the possibility of acute stroke. The data were unselected except for the presence of radiologist-reported changes on diffusion weighted imaging consistent with ischaemic stroke and a minimum lesion volume of 216 mm^3^. Note that this volume, corresponding to damage occurring to a 6 mm^3^ volume of brain tissue, is much smaller than the lesions generally used in lesion mapping studies and so is unlikely to be a source of the bias that we demonstrate is related to the disparity between lesion volume and functionally critical volume. The mean age was 62.3 years, standard deviation (SD) = 17.8, and the proportion of males was 0.547. The data for each patient consisted of standard, axially-acquired diffusion-weighted echoplanar imaging (b0 and b1000) sampled at 1 × 1 × 6.5 mm resolution, and obtained on a GE Genesis Signa 1.5 T MRI scanner in a single session for each patient. The b0 images satisfactorily distinguish between CSF, grey and white matter in line with their T_2_-weighting while being relatively unaffected by acute ischaemic lesions. The b1000 images, by contrast, show little normal tissue differentiation but marked differences in signal between damaged and undamaged tissue. The complementarity between these two sequences is exploited in the subsequent processing described below. The study was approved by the local ethics committee.

### Image preprocessing

To make comparisons between the lesions of different people, we first co-registered each brain image to the same standard template so that anatomically homologous regions are brought into alignment, a process commonly referred to as image normalization. We then distinguished lesioned from normal brain through automated lesion segmentation. Here we followed previously evaluated methodology described in detail elsewhere (Mah *et al.*, 2012), implemented in SPM8 (http://www.fil.ion.ucl.ac.uk/spm/software/spm8/) and custom MATLAB (http://www.mathworks.co.uk/products/matlab/) scripts. In brief, we first co-registered each b0 image to its b1000 counterpart using SPM8’s rigid body co-registration routine. This ensured that transformation parameters derived from the b0 image could subsequently be applied to the b1000. We then normalized each b0 image using SPM8’s combined tissue segmentation/normalization routine, including an extra tissue class in the mixture model to minimize distortion from any abnormal signal in the b0 (Crinion *et al.*, 2007). Because the lesions were all acute, signal abnormalities on the b0 were generally minor. The normalization parameters so derived were then applied to each b1000 image, bringing it into standard stereotactic space, resliced to 2 × 2 × 2 mm resolution. The b1000 images now being co-registered so that homologous regions were in alignment, we were able to apply a voxel-wise, automated lesion segmentation algorithm optimized for diffusion weighted imaging, and shown to perform comparably to the current gold standard: manual lesion segmentation (Mah *et al.*, 2012). The output of this step for each patient was a binary image, indexing the presence or absence of damage to any part of the brain, at 2 × 2 × 2 mm resolution. Note that the automated lesion-segmentation algorithm cannot be a significant source of the effects we observe in the data because it is inherently agnostic of the vascularity-informed pattern that is shown to dominate them. An overlap map of all normalized lesion masks is shown in Supplementary Fig. 1.

The rare case of anomalous anterior cerebral artery circulation aside, acute ischaemic strokes are unilateral affecting one hemisphere only. Even when (rarely) ‘showers’ of emboli cause multiple strokes, damage in each hemisphere is generally independent of the other. As there are also no reported consistent differences in the anatomy of the vascular tree between the two hemispheres, we therefore collapsed the data onto one hemisphere, choosing, for each patient, the hemisphere with the largest (most commonly the only) lesion, and correspondingly flipping the image volume in the mid-sagittal plane. Note that any consistent lateralized anatomical differences between the two hemispheres would tend to reduce the inhomogeneity of the resultant data set (for the stereotypy of the underlying patterns would be reduced) and so this manipulation could only reduce the size of any effect we demonstrate, not increase it or spuriously create it. All subsequent analyses were performed on these single hemisphere binary images.

### Assessing the impact of high-dimensional lesion pattern inhomogeneities on lesion mapping

To determine how the conventional methodology of lesion mapping affects the fidelity of the result we need to know the true neuroanatomical dependence of any putative function—something we do not know, and cannot know, until we have precisely the model of the brain for which we need lesion mapping in the first place. How do we get around this impasse? We can build a set of hypothetical models of lesion-deficit dependence with parameters that make them less susceptible to bias than physiologically plausible models. If such artificially benign models show significant error then we know the reality can only be worse. Though inevitably a model, our inference then has generality over reality.

We began with the simplest possible model that can be evaluated with our data set: single voxel dependence of a putative function of interest. For each of 90 469 models, corresponding to each voxel location hit at least four times in the data set, a single voxel location in the brain was taken as being critical to a hypothetical deficit. We then labelled each of the 581 scans in our data set as being ‘affected’ or ‘unaffected’ depending on whether or not that voxel fell within the lesion present in the scan. This gave us a simulated ‘ground truth’ label for the model, splitting the set into two groups, just as if they were two patient groups differing in behaviour or some other outcome. We then ran a mass-univariate analysis at each voxel, treating each voxel independently of every other, producing a voxel-wise *P*-value map across the brain testing the null hypothesis that the voxel is unrelated to the group label. The statistical test was Fisher’s exact test, a non-parametric test widely used in this setting, deriving the asymptotic *P-*value (Fisher, 1970). The resultant *P*-map was thresholded at *P < *0.01, Bonferroni corrected for multiple comparisons. This yielded a significant cluster of voxels, inevitably including the voxel defining the label, but not necessarily centred on it. We then identified the centre of mass of the significant cluster and calculated its displacement from the label-defining voxel, giving us a vector value at that voxel quantifying the direction and magnitude of the error introduced by the mass-univariate technique. We repeated this modelling process for each of the 90 469 voxels in our data set that was affected in at least four of the set of 581 scans, resulting in an ‘error vector map’ across the brain giving the direction and magnitude of the error at every voxel tested. This map was visualized using ParaView (http://www.paraview.org/), the vector at each voxel within illustrative axial, coronal, and sagittal slices represented by a tail-less arrow with a magnitude and direction given by the displacement, and colour given by the direction within the plane being illustrated. Summary measures of the absolute error were calculated by taking the mean of the Euclidean distances, across voxels, and their standard deviation.

Single voxel dependence is too simple a model to be plausible biologically. Lesions of the volume occupied by one voxel in our data set rarely produce unique symptoms clinically unless they fall within critical subcortical or brainstem regions. A more plausible model is one where a deficit is sensitive to damage to a subset of a spatially extended cluster of functionally related voxels. The most convenient *a priori* clustering we can use here, for cortex at least, is the Brodmann map. We therefore constructed a further set of models where the ‘ground truth’ was defined not by the presence of damage to a single voxel but the presence of damage to at least 20% of any of the voxels falling within a given Brodmann area (BA) and its underlying white matter as defined in the template distributed with MRIcro software (http://www.mccauslandcenter.sc.edu/mricro/index.html). Note that the precise anatomy of the area parcellation here is not critical, for we do not know what the critical functional parcellation really is. We simply need to explore the consequences of an area-based model, and this is as good a parcellation as any other. Further, to increase the biological plausibility of the model we made the relation between the presence of a lesion and the label of ‘affected’ stochastic rather than deterministic, with a probability of 90%. We evaluated such a model for each Brodmann area, performing the mass-univariate inference otherwise exactly as in the single-voxel models. For completeness, we evaluated further variants of this model, where the critical threshold for designating a lesion as ‘affected’ was varied, in separate models, from 5% to 60%, in 5% bins. Note that as there are no empirical data on the proportion of any given functionally homogeneous area that needs to be inactivated for the area as a whole to malfunction, these variants are intended purely to show that the result at the 20% threshold is not an accident of that specific choice. The displacement in the estimate of the location of each Brodmann area was calculated as the difference between the centre of mass of the corresponding Brodmann area and the centre of mass of the cluster identified by the mass-univariate test as significant. Summary measures of the absolute error were calculated by taking the mean of the Euclidean distances, across voxels, and their standard deviations.

Where more than one voxel crosses the significance threshold, choosing the centre of mass of the significant cluster is an established approach for reporting the localization in lesion studies, either explicitly or implicitly by referring to the anatomical region where the largest proportion of the significant cluster falls (Karnath *et al.*, 2001, 2011; Mort *et al.*, 2003; Golay *et al.*, 2008). Within the traditional frequentist statistical framework, the null hypothesis that any of the voxels with a *P*-value lower than the threshold are not related to the deficit should be rejected, with no grounds to give preference to one voxel over another within the significant set. By that established standard, the centre of mass is as good a measure as any. However, it may be argued that this aspect will itself introduce a distortion, sensitive to the anatomical location of the boundaries of the significant region rather than its peak. As the boundaries depend on the strength of the relation between a lesion and the presence of a deficit—an unknown and ungeneralizable factor—the correct degree of distortion is difficult to model. We therefore evaluated exactly the same model as above, but this time calculating the error as the vector difference between the peak significant voxel and the centre of mass of the target Brodmann area. The summary measures of the absolute error were calculated as above.

Now although connectivity in the brain is dominated by local connections, it is clear that areas that are anatomically remote may nonetheless be functionally related. Models that ignore such long-range connections are biologically implausible. To assess the impact of such distributed neural dependence we explored what happens when damage to either of two clusters across the brain is present. Combinatorial expansion makes comprehensive modelling of this computationally intractable, but we can nonetheless take an illustrative pair of areas. A comprehensive evaluation would be unhelpful in any event, as we do not know that the Brodmann parcellation, or any known parcellation, is truly representative of the underlying distributed functional anatomy. The critical point is that if a problem can be demonstrated for one biologically plausible pairing, then no hypothetical pairing can be trusted.

Here we therefore evaluated a model where damage to ≥20% of either BA 39 or BA 44—two putative loci for visuospatial neglect—causes a hypothetical deficit of interest 90% of the time. A mass-univariate analysis based on this label, carried out exactly as above, places the critical locus mostly outside the two true areas, with the centre of the cluster of activation in the superior temporal gyrus, within a wholly different lobe of the brain. Because the actual level of significance is here immaterial—the hypothetical model being artificial—we show for display purposes a threshold yielding the same number of surviving voxels as there are voxels in BA 39 and BA 44 combined.

As a further illustration of the generality of such mislocalization, we repeated the same analysis, keeping all model parameters the same except now defining as hypothetically critical BA 38 and BA 37: two distant loci implicated in picture naming (Price *et al.*, 2005; Hillis *et al.*, 2006; Schwartz *et al.*, 2009; Trebuchon-Da Fonseca, *et al.*, 2009; Baldo *et al.*, 2013). The pattern of displacement will naturally vary depending on the number and location of critical Brodmann areas chosen, and so testing further pairings is unhelpful as one could explore only a small subset of all possible combinations. It suffices to show substantial error for two plausible pairs to throw any combination into doubt.

### High-dimensional multivariate inference

The next question we addressed was whether or not methodology that attempts to capture the high-dimensional multivariate distribution of lesion damage can overcome these difficulties. Again, a comprehensive evaluation is impossible because the combinatorial possibilities are too great in number. Nonetheless, we can compare the performance of a high-dimensional multivariate approach in the same examples as the preceding two-area simulations: as the mass univariate approach fails here, success with multivariate modelling would show that the approach is at least worth pursuing, even if never guaranteed to succeed.

We therefore remodelled both the BA 39/44 (for neglect) and BA 37/38 (for picture naming) examples above in exactly the same way except that the inferential test was applied not at each voxel but for each brain volume, each voxel now being treated as a variable or dimension rather than a single variable in a univariate statistical test independently replicated across voxels. Each case in this high-dimensional multivariate model was thus specified by 90 469 binary variables (the predictor variables, in machine learning jargon) indexing the presence or absence of damage across the entire brain for that specific case, and one binary variable (the target variable) indexing the presence or absence of a hypothetical deficit in that specific case, determined by the putatively critical regions exactly as in the preceding mass univariate example.

To estimate such a model, we cannot use conventional statistical techniques such as multivariate ANOVA because there are too many variables in proportion to the number of cases. Instead, we must use a statistical classifier based on supervised machine learning, where the model is fitted and evaluated by iterative training and testing on independent, randomly selected subsets of the data. In the training phase, the classifier is trained on a subset of the data (using both predictor and target variables) so as to find the maximal separation in the high-dimensional space defined by the predictor variables of the groups defined by the target variables. The performance of the trained classifier is then tested against an independent subset of the data, comparing its predicted target variables with the known target variables for that subset. The procedure is iterated for two purposes: first, to derive confidence measures of classifier performance across different partitionings of the data, thereby minimizing the risk of a spurious result from accidental overfitting, and second, to tailor the parameters of the classifier so as to optimize it for the specific task.

What such a classifier learns is to be able to discriminate between two groups within a high dimensional data set. How it makes the discrimination may be opaque, depending on the type of classifier used. But certain types of classifier yield weights for each predictor variable, allowing us to compare their relative contribution to the discrimination process, rather as one does with the weights in a conventional logistic regression model. Though not explicitly testing the hypothesis of the criticality of each variable, the value of these estimates is corroborated by the prediction performance of the model overall. If the model is highly sensitive and specific in its predictions, then the weights ought to capture the relative contribution of the variables well.

In this study, we used a multivariate support vector machine classifier with a linear kernel (http://www.csie.ntu.edu.tw/∼cjlin/libsvm/), iteratively training and testing on subsets of the data while modifying the kernel *C* parameter so as to optimize the fit as estimated by classification performance. The *C* parameter evaluation was performed across the range from *C* = 2^−20^ to *C* = 2^20^. This was done by taking, 17 times, randomly chosen subsets of 556 cases, training the classifier on these, and testing on the remaining 25. Once optimally trained, the weights assigned to each dimension (i.e. voxel) were used to index its contribution to the classification process, giving us a measure of the estimated importance of each voxel for the deficit being modelled. As there is no established way of interpreting the significance of these weights (such as a criterial threshold), to compare the performance of the multivariate and mass-univariate models we thresholded the weights so as to yield the same number of surviving voxels in the multivariate model as were present in the mass-univariate one. Although only suggestive, showing that the multivariate approach may succeed where the mass univariate approach definitely fails is good grounds for considering a shift in policy on methodology. The final performance of the trained model was evaluated without any noise in the testing data (i.e. with 100% correspondence between the lesion criterion for a ‘deficit’ and its assignment as the label), for otherwise we would be unhelpfully adding noise to our estimate of how well the model has been trained as well as to the model training itself.

## Results

It is crucial to recognize that the extent of mislocalization resulting from a hidden bias within the multivariate distribution cannot be shown by examining an example lesion-deficit relationship within the data set because such an analysis would be viciously circular: we only have lesion-deficit mapping to determine the real locus from which any erroneous localization may deviate; there is no other standard to appeal to. Instead, we must create a large array of hypothetical lesion-deficit models where the consequences of a given locus being critical in reality are explicitly tested within the data set, and the process is iterated over the widest possible range of loci.

We start by positing a locus and labelling each image in our data set of 581 images as being ‘affected’ or ‘unaffected’, dependent on whether or not the locus falls within a lesion in that image. For example, when a locus in inferior frontal gyrus is chosen, all brains in which that locus falls within the lesion are labelled as being ‘affected’ and all those where that locus falls outside the lesion are labelled as being ‘unaffected’. This creates two hypothetical patient groups defining the ‘ground truth’ in the model as determined by the posited locus. We next use the ground truth label to perform a standard mass-univariate statistical analysis across all voxels in the brain, just as one would in a lesion-deficit mapping study except, crucially, that here the true locus is known. The locus inferred from this analysis is then compared with the true locus to quantify any error as a vector pointing from the true locus to the inferred one. If the collateral damage associated with a given locus is random, i.e. if there is no hidden bias in the multivariate distribution of damage, the error would not show a consistent direction and would average out at zero. Conversely, if a hidden structure is present, a consistent error would be shown, with a magnitude and direction dependent on the nature of the underlying multivariate distribution.

**Figure 2:**
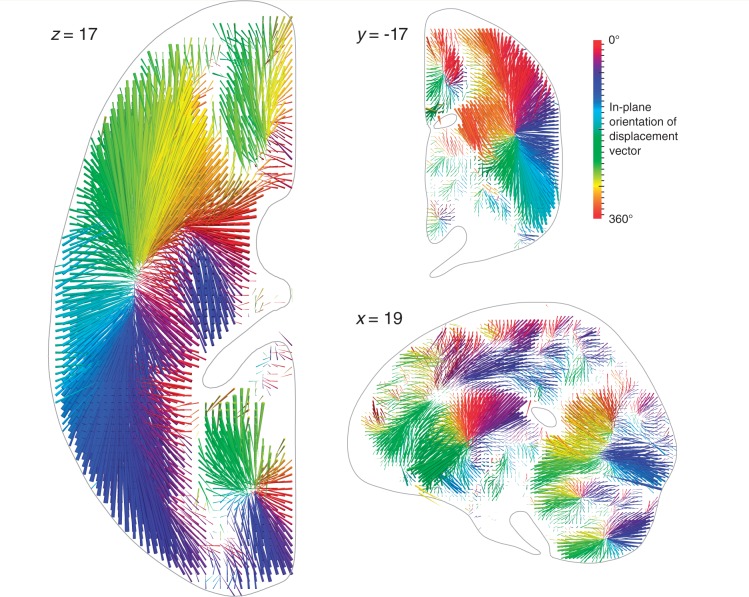
Three-dimensional vector plot of the direction (colour map) and magnitude (length of arrow) of mislocalization at adequately sampled voxels within three representative planes (*left* axial, *top* coronal, *bottom* sagittal), based on a sample of 581 acute stroke lesions, normalized into standard stereotactic space and mirrored onto one hemisphere (see ‘Materials and methods’ section for details). The value at each voxel was calculated by labelling the stack of 581 lesioned volumes as being ‘affected’ or ‘unaffected’ depending on whether or not that voxel fell within the lesion in each volume, running a standard voxel-wise Fisher’s exact test-based mass-univariate analysis on the two groups, and identifying the centre of mass of the resultant significant cluster, identified by the asymptotic *P*-value thresholded at a Bonferroni corrected *P < *0.01. This procedure was performed at all voxels hit more than three times in the data set. Each arrow points from the true location of a voxel in the brain to the location where the mass-univariate model erroneously places it. The colour map corresponds to the orientation of this error vector in the visualized plane. Note that the mislocalization tends to follow the organization of the vascular tree, with clusters corresponding to the branches of the middle cerebral, anterior cerebral, and posterior circulations. See ‘Materials and methods’ section for details. See Supplementary material for manipulable 3D versions of these images.

Initially, to determine the minimum size of any such systematic error we first modelled the simplest possible lesion-deficit relation: a single voxel locus with a one-to-one mapping between damage to the voxel and the loss of a function exclusively dependent on it. Iterating over every adequately sampled voxel location in our data set—a total of 90 469 models—we generated a comprehensive ‘mislocalization map’ (see ‘Materials and methods’ section for details). This map is a vector field describing the magnitude and direction of the displacement from the true locus at each voxel, were that voxel to be critical for a given function (Fig. 2, data collapsed onto one hemisphere for simplicity; see Supplementary material for manipulable 3D version of the plots).

Very substantial mislocalization was observed in all regions of the brain, with a mean of 15.7 mm (SD = 9.15 mm). This is more than sufficient to mislocalize across lobes of the brain, from one Brodmann area to another, or from grey to white matter. Crucially, the direction of the error was not random, but qualitatively followed the architecture of the underlying vascular tree, resulting in consistent patterns of mislocalization: most prominently a shift of cortical loci to deep white matter, and a shift of frontal, temporal, and parietal loci to the vicinity of the path of the middle cerebral artery and its branches. Such consistent mislocalization is naturally impossible to remedy through replication alone because it emerges from the intrinsic neurovascular architecture of the brain, and can only be reinforced by repetition.

Our initial models hypothesize an unrealistically simple relation between anatomy and deficit, where functions are localized to single voxels. In such circumstances the inferred locus will always contain the critical voxel, though of course the centre of the cluster of voxels crossing the significance threshold could be, and in our models is, located elsewhere, distorting the inference. If the relation between damage and loss of function is more complex than this, the mislocalization is likely to be greater. Specifically, if as in the example in Fig. 1, damage to any part of a critical region can result in dysfunction of the whole region the maximally significant locus identified by conventional lesion mapping need not even include any part of the critical region.

Next, to test this more physiologically plausible model we repeated the analysis with the hypothetically critical loci now being not individual voxels, but groups of voxels falling into standard Brodmann areas and their immediately underlying white matter. In each model, we posited damage to an area associated with a deficit in a given brain if ≥20% of its constituent voxels were affected. To further increase physiological plausibility we also made the relation between damage and ‘deficit’ not deterministic but stochastic, with a 90% probability of a deficit when the damage criterion was met. Independent models were created for each Brodmann area to generate another mislocalization map for this more biologically realistic relationship between anatomy and function.

A large displacement was also observed here—mean 15.9 mm—with greater variability across the brain: SD = 17.6 mm. The displacement was not substantially attenuated by calculating the error as the difference between the location of the Brodmann area and the peak, rather than the centre of mass, of the estimated cluster: the mean displacement here was 15.2 mm (SD = 18.2 mm). Equally, the average of mean displacements across a range of critical volume thresholds—from 5% to 60%—was 13.6 mm (SD = 3.1 mm), showing that the magnitude of the observed error is not an artefact of the 20% threshold.

Quantifying the mislocalization in this way may potentially provide the means of eliminating it, e.g. by applying the inverse of the error vector field (Fig. 2) to any lesion-deficit mapping study. However, such correction would be valid only if the underlying lesion-deficit model is valid: in particular, if the function is dependent on a single, voxel-sized locus. Given the complex, distributed organization of the brain this is not a plausible assumption to make. We must therefore also determine the error with models where a given function is dependent on multiple loci, including those that are spatially non-adjacent. This cannot be achieved comprehensively across the brain because the vast number of combinatorial possibilities makes it computationally intractable. But if a substantial error is present in one, physiologically plausible example, other possibilities would be comparably imperilled.

Indeed the controversy over the locus of visuospatial neglect following focal brain injury offers a striking example (Shirani *et al.*, 2009; Molenberghs *et al.*, 2012; Smith *et al.*, 2013; Vuilleumier, 2013). Though some lesion evidence and physiological plausibility derived from functional imaging have strongly suggested two independent candidate areas—BA 44 and BA 39 (Husain and Kennard, 1996; Mort *et al.*, 2003; Verdon *et al.*, 2010), another lesion study placed the locus in a single region: the superior temporal gyrus (Karnath *et al.*, 2001, 2004). To examine the possibility that these discrepant localizations may have been a consequence of the distortion we have identified, we created a model where damage to ≥20% of either BA 44 or BA 39 was hypothetically critical in 90% of cases (see ‘Materials and methods’ section for details).

**Figure 3:**
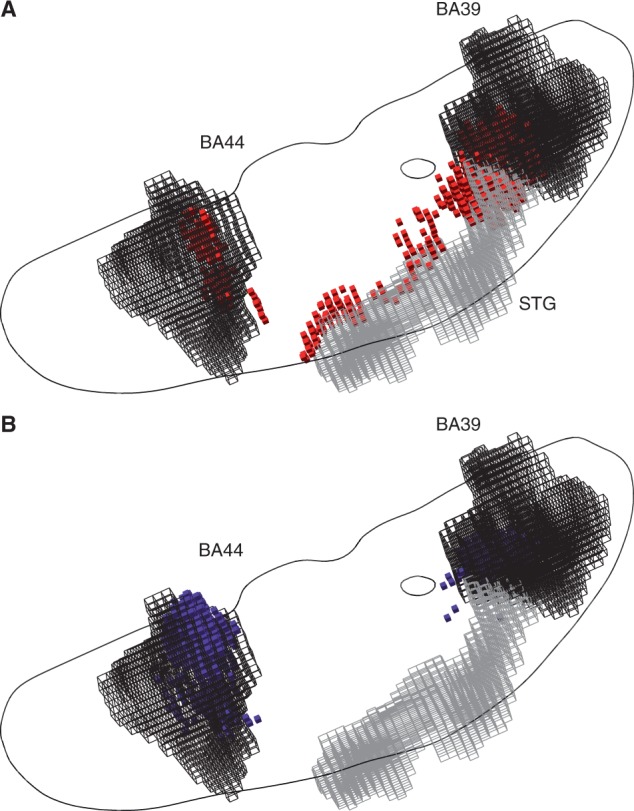
(**A**) Three-dimensional plots of the voxels identified as significantly associated with a hypothetical deficit—given damage to either BA 39 or BA 44 at ≥20% of the volume of either—by a voxel-wise mass-univariate analysis of the sample of 581 acute stroke lesions (red cubic glyphs). As before, Fisher’s exact test was used, thresholded at a level such that the volume of surviving voxels equalled 20% of the volume of BA 39 and BA 44 (each area is shown as a black wireframe). Note that the centre of mass of the significantly associated region falls in neither Brodmann area, but in the region of the superior temporal gyrus (STG, grey wireframe). See ‘Materials and methods’ section for details. In grey is an outline of an axial slice traversing BA 44 and BA 39, shown here purely to give an indication of the relative position of the two areas in the axial plane. (**B**) Three-dimensional plots of the voxels identified as heavily weighted in the classification process—given damage to either BA 39 or BA 44 at ≥20% of their total volume—by a high-dimensional multivariate analysis of the sample of 581 acute stroke lesions based on a linear support vector machine (blue cubic glyphs). The voxels shown are thresholded so as to yield the same number of surviving voxels as in the mass univariate analysis depicted in **A**. Note that the mislocalization observed with the mass-univariate approach is no longer seen. See ‘Materials and methods’ section for details. See Supplementary material for manipulable 3D versions of these images.

Strikingly, the model showed a substantial erroneous displacement of the inferred critical region to the superior temporal gyrus. The results reported by Karnath *et al.* (2001) are therefore consistent with the critical region not being the superior temporal gyrus, but damage to either BA 44 or BA 39 (Fig. 3A, see Supplementary material for a manipulable 3D version of the plot). Note that our results do not imply that BA 39 and BA 44 are necessarily critical; only that a combination of two critical areas other than superior temporal gyrus may artefactually mislocalize there when mass-univariate inference is used.

**Figure 4:**
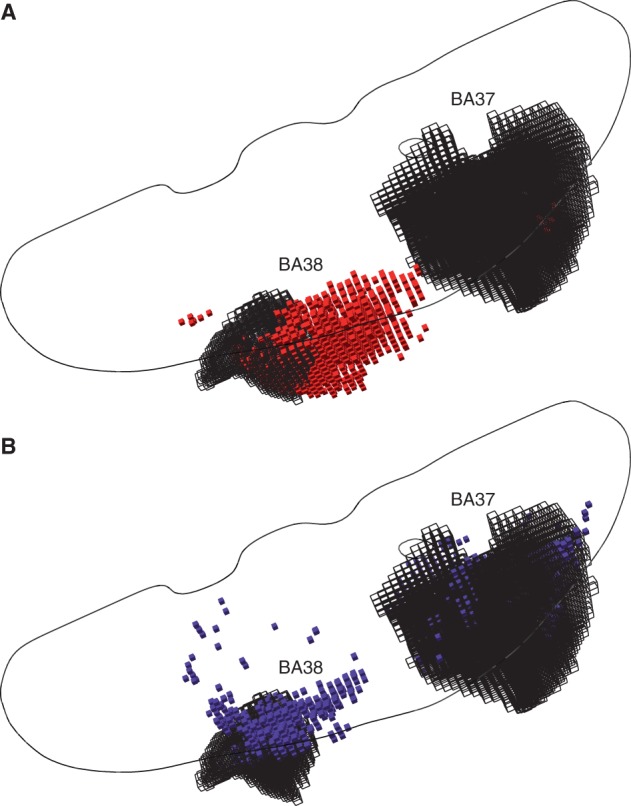
(**A**) Three-dimensional plots of the voxels identified as significantly associated with a hypothetical deficit—given damage to either BA 37 or BA 38 at ≥20% of the volume of either—by a voxel-wise mass-univariate analysis of the sample of 581 acute stroke lesions (red cubic glyphs). As before, Fisher’s exact test was used, thresholded at a level such that the volume of surviving voxels equalled 20% of the volume of BA 37 and BA 38 (each area is shown as a black wireframe). Note that the centre of mass of the significantly associated region falls in neither Brodmann area. See ‘Materials and methods’ for details. In grey is an outline of an axial slice as in Fig. 3, shown here purely to give an indication of the relative position of the two areas in the axial plane. (**B**) Three-dimensional plots of the voxels identified as heavily weighted in the classification process—given damage to either BA 37 or BA 38 at ≥20% of their total volume—by a high–dimensional multivariate analysis of the sample of 581 acute stroke lesions based on a linear support vector machine (blue cubic glyphs). The voxels shown are thresholded so as to yield the same number of surviving voxels as in the mass univariate analysis depicted in **A**. Note that the mislocalization observed with the mass-univariate approach is much less pronounced. See ‘Materials and methods’ section for details. See Supplementary material for manipulable 3D versions of these images.

To take another example, the locus of picture naming shows a surprisingly varied distribution, ranging from anterior temporopolar to lateral posterior temporal cortex (Price *et al.*, 2005; Hillis *et al.*, 2006; Schwartz *et al.*, 2009; Trebuchon-Da Fonseca, *et al.*, 2009; Baldo *et al.*, 2013). Here we modelled BA 38 (temporal pole) and/or BA 37 (lateral posterior temporal cortex) as hypothetically critical loci. The inferred critical region is here also substantially displaced, with a striking imbalance in favour of BA 38, despite its smaller volume, probably owing to the reduced anatomical variability of patterns of damage close to the course of the middle cerebral artery (Fig. 4A, see Supplementary material for a manipulable 3D version of the plot). It is easy to see how a wide variety of erroneous patterns may be generated here, spanning a large swathe of cortex and white matter wholly outside the actual critical loci.

Crucially, these errors occur not because the data lack the spatial resolution to distinguish between the two areas correctly but because the conventional inferential approach cannot handle its complexity. Evaluating exactly the same models with a high-dimensional multivariate approach based on a linear support vector machine allowed us both to predict the target variable with high fidelity [BA 39/44: sensitivity 0.804 (SD = 0.117), specificity 0.968 (SD = 0.046), BA 37/38: sensitivity 0.8580 (SD = 0.12), specificity 0.952 (SD = 0.061)] and successfully identifies the two critical areas by the distribution of weights assigned to the constituent voxels by the classifier (Figs 3B and 4B, see Supplementary material for a manipulable 3D versions of the plots).

## Discussion

These analyses demonstrate a crucial feature of the data: the pattern of mislocalization across the brain will depend on the complex interaction between the multivariate lesion distribution and brain functional architecture. As the latter is unknown, indeed precisely what we are using lesion mapping to establish, we need to know the former to have any confidence in our predictions. Replications with larger numbers of cases cannot reduce the extent of mislocalization, for the problem arises not from random error but from consistent biological biases that increased numbers can only amplify. Indeed, as our data set is an order of magnitude larger than most lesion-deficit mapping studies, even meta-analytic analyses could not compete numerically.

It is tempting to object that models of lesion-deficit associations based on lesion data alone cannot tell us anything definitive without incorporating real deficits. This is incorrect for three reasons. As with any complex empirical experiment, the final error in any lesion study will be the sum of component errors. Here we have at least two component errors: one resulting from the nature of the high-dimensional lesion distribution, and another from the nature of the lesion-deficit association. As the component errors are additive, if the first is substantial—as we have shown here—then the size of the second can only increase the error, not reduce it. Indeed, our models assume a fidelity of lesion-deficit association that is likely to be higher than any obtaining in reality. A model that incorporates deficit as well as lesion data can therefore only show greater error.

Second, in the absence of a certain ‘ground truth’ that another empirical method can securely determine, one cannot reliably estimate the lesion component error without positing a hypothetical ground truth for the lesion-deficit association. If a putatively better empirical method—for example the high dimensional inference we propose below—also mislocalizes, though to a lesser degree, the size of the overall error will be unquantifiably concealed.

Third, a crucial finding here is the substantial variation across the brain of the relation between the specific functional locus and the resultant mislocalization: one can therefore say nothing general about the size and direction of the error from a study of any one deficit or locus. Indeed, attempting to do this would be misleading, for the reasons we have already given.

It may also be objected that there are simple parameters of the pattern of damage, such as total lesion volume, that one could use to try to correct inferential models that remain fundamentally mass-univariate. Indeed, lesion volume is routinely parameterized in lesion studies. Far from correcting the distortion, such an approach would add further distortion from the likely complex high-dimensional multivariate relation between lesion volume (or any other reductive parameter) and the lesion pattern. For example, since lesions involving the cortex are more commonly larger in volume than those confined to subcortex, cortical regions will be unfairly penalized (Husain and Nachev, 2007).

Equally, a richer parameterization of the functional deficit—for example by a numbered score rather than a binary measure—cannot change an effect fundamentally driven by correlations across the predictor variables; indeed in conditions where the deficit can be graded there is an added complexity of residual partial function and its anatomical dependence that can only add to the error, though whether systematically or not would need investigation case by case.

We propose three practical solutions. The first is to confine the use of lesion-deficit mapping to choosing between predefined functional anatomical models derived from an experimental modality less prone to spatial bias, such as functional imaging. This is unsatisfactory because the alternative modality need not correctly identify the pattern of functionally necessary areas (necessity—evaluated comprehensively across the brain—being precisely what we need lesion-mapping for). The second is to use small, non-overlapping lesions, with minimal collateral damage. Though potentially powerful and an important area for future development (Editorial, 2004), this approach is hampered by the low natural frequency of such lesions, and their predilection for particular areas, limiting the feasibility of studies with adequate patient numbers.

The third and only comprehensive solution is to construct lesion-deficit models using high-dimensional inference that captures the multivariate lesion distribution, explicitly modelling the parasitic voxel-voxel associations that are the source of the error. We have seen that re-analysing our two-area simulations with the aid of such high-dimensional inference successfully separates the two critical areas (Fig. 3B). Where this methodology can capture comprehensively the pattern of damage to the brain as a whole—not an easy task—a correct structure-function mapping can theoretically be achieved.

This is not a panacea, however. Estimating such models requires non-traditional inferential methods based on machine learning (MacKay, 2003). Owing to the very large number of variables it also requires much larger numbers of cases (hundreds to thousands) than is usual in the lesion mapping literature. The exact size will be known once the lesion distribution has been sufficiently well characterized: large-scale, collaborative lesion databases may be one approach to making such determinations. Although our multivariate model works for two areas, this does not necessarily imply that more complex relationships between damage and dysfunction will allow the method to work for multiple critical areas. Furthermore, any test—whether multivariate or univariate—can only distinguish between two models to the extent to which the variance in the data can be captured better by one model or the other. If an irrelevant single variable happens to be better correlated with the outcome than any complex pattern, for whatever reason, then multivariate inference cannot help because the problem is simply not soluble with the data at hand.

But whereas current lesion mapping practice cannot be corrected by replication with greater numbers, the multivariate approach we propose here can, and ought ultimately to converge on the true locus (or loci) in each case, as far as the data allows. It is outside the scope if this study to determine the optimal multivariate approach: our focus here is on the evidence of the misleading picture the mass-univariate approach has created, and the need to review it wholesale. Taken together, our work demonstrates a way forward to place the study of focal brain lesions on a robust theoretical footing. This will allow localizations we have shown to be insecure to be revisited with a methodology that is resistant to the critical errors we have empirically identified.

## Funding

This work is funded by the Wellcome Trust (Grant number ME049685) and the UCLH NIHR Biomedical Research Centre.

## Supplementary material

Supplementary material is available at *Brain* online.

Supplementary Data
