# Supplementary material for: Human brain lesion-deficit inference remapped
Source: Brain. 2014 Jun 28;137(9):2522–31. doi: 10.1093/brain/awu164 (PMC4132645; doi:10.1093/brain/awu164)
Supplement: Supplementary Data [file supp_137_9_2522__index.html]

Human brain lesion-deficit inference remapped — Supplementary Data 

# Human brain lesion-deficit inference remapped

## Supplementary Data

files

**Files in this Data Supplement:**

- Supplementary Data - pdf file
- Supplementary Data - pdf file
- Supplementary Data - pdf file
- Supplementary Data - pdf file
- Supplementary Data - pdf file
- Supplementary Data - pdf file
- Supplementary Data - pdf file
- Supplementary Data - pdf file
