# Supplementary material for: Human brain lesion-deficit inference remapped
Source: Brain. 2014 Jun 28;137(9):2522–31. doi: 10.1093/brain/awu164 (PMC4132645; doi:10.1093/brain/awu164)
Supplement: Supplementary Data [file supp_awu164_brain-2014-00069-File003.pdf]

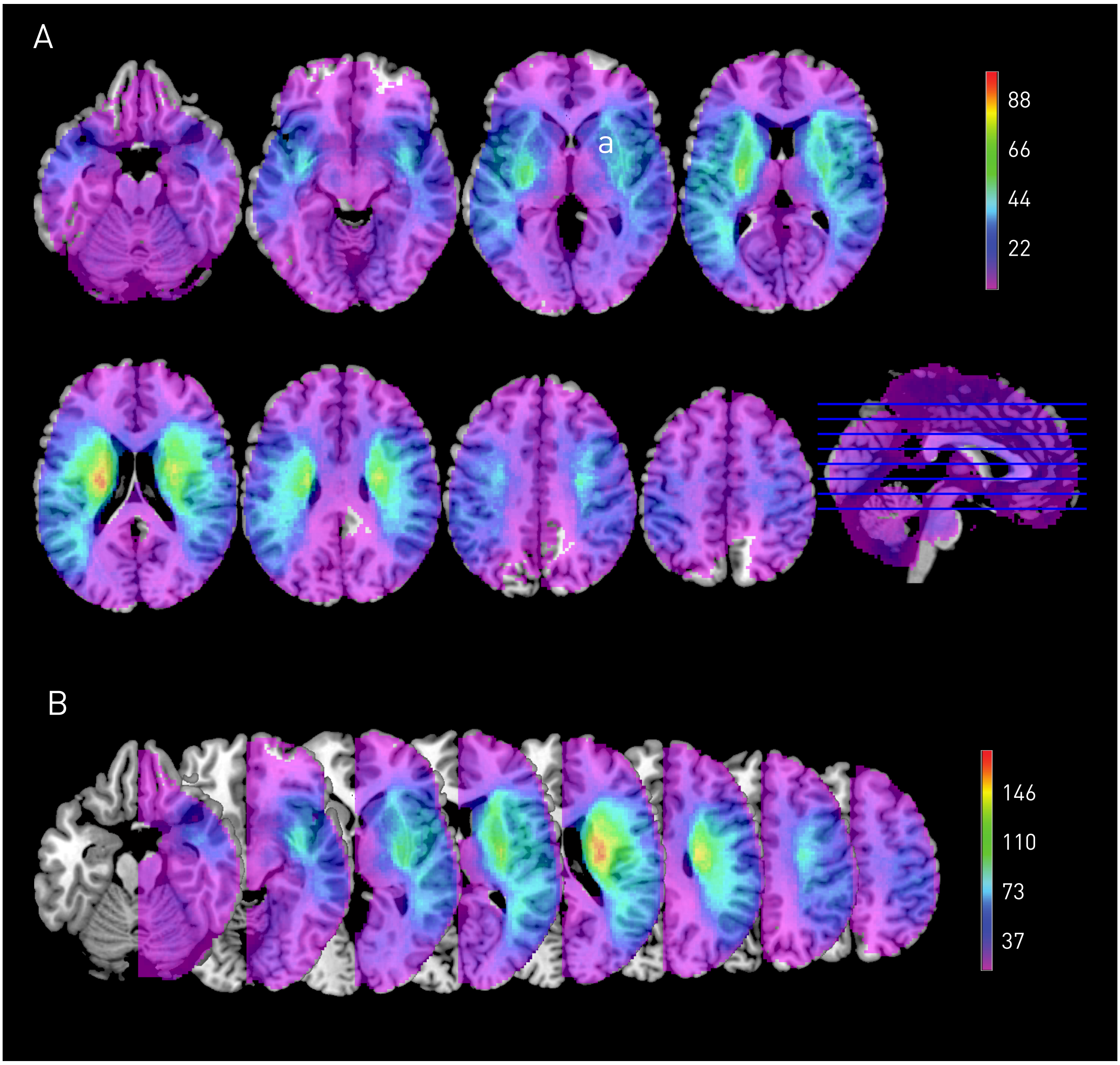

Supplementary Figure 1. A. Lesion overlay of all 581 lesion masks used in the study, with a colourmap indicating the absolute numbers at each voxel. The maximum hit rate was 96 per voxel. The axial slices are located at z=-20, -10, 0, 10, 20, 30, 40 and 50mm in MNI space. Note that the distribution of lesion injury is essentially symmetrical across the mid-sagittal plane. B. Lesion overlay of all 581 lesion masks collapsed onto the right hemisphere, as used in the study. The axial slices are located as before. The peak overlay here is 160 per voxel. Note that the colourmap has been adjusted to the new range.
