# Supplementary figures and images for: Human brain lesion-deficit inference remapped
Source: Brain. 2014 Jun 28;137(9):2522–31. doi: 10.1093/brain/awu164 (PMC4132645; doi:10.1093/brain/awu164)

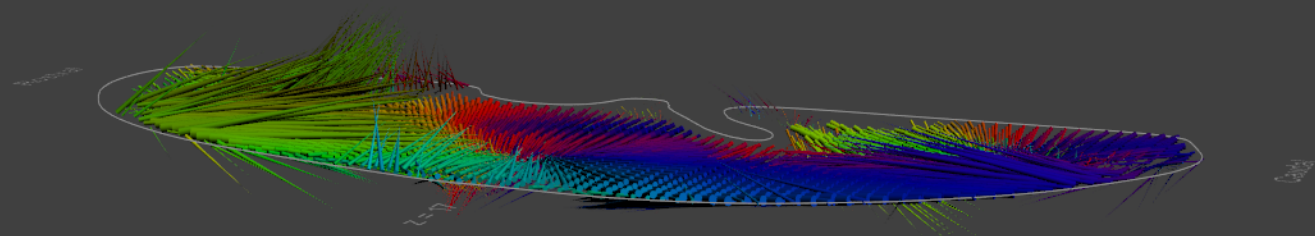

Supplement: Supplementary Data [file supp_awu164_brain-2014-00069-File004.pdf]

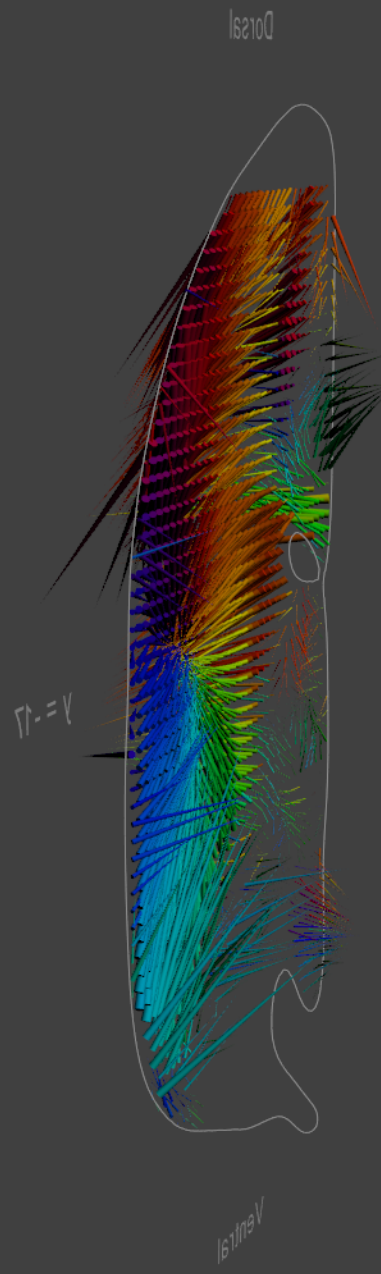

Supplement: Supplementary Data [file supp_awu164_brain-2014-00069-File005.pdf]

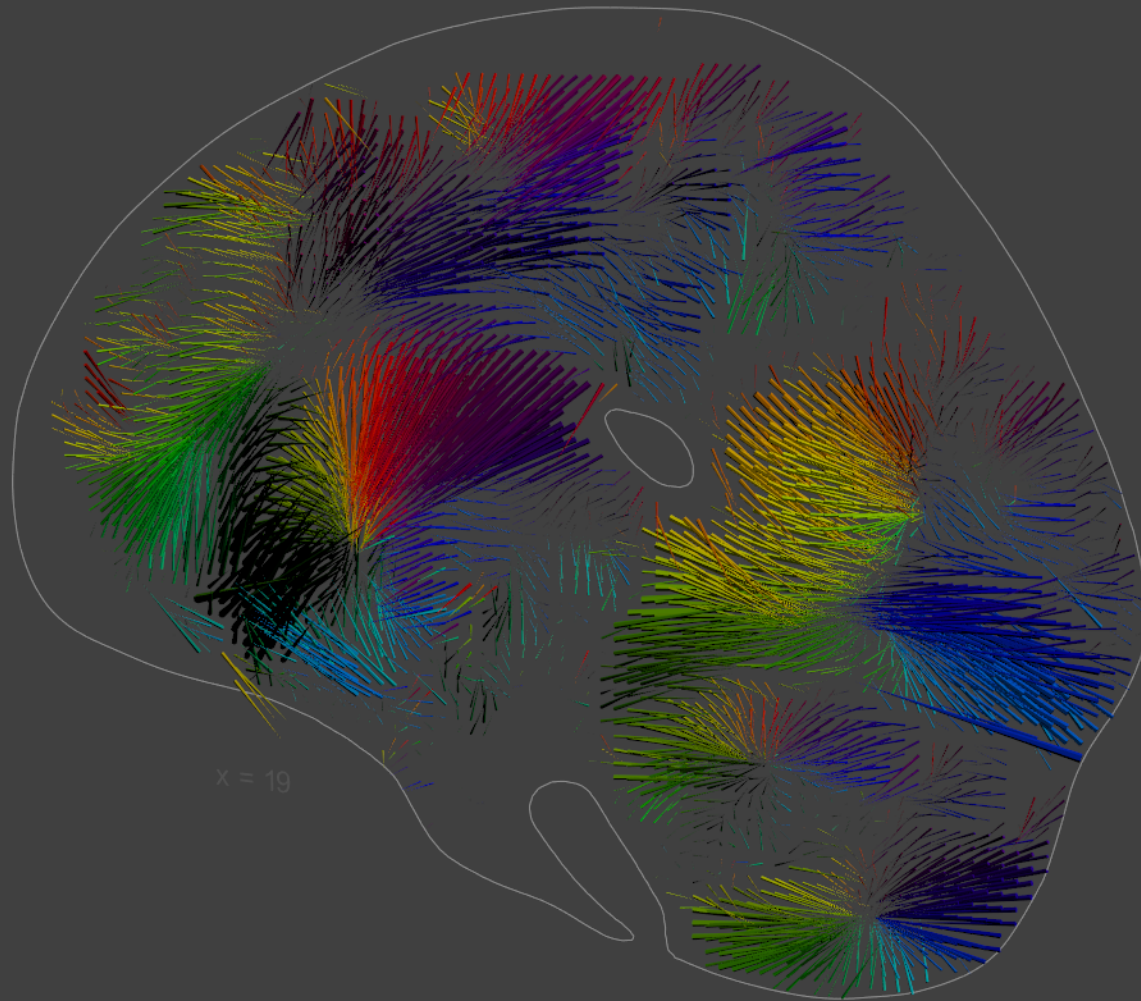

Supplement: Supplementary Data [file supp_awu164_brain-2014-00069-File006.pdf]

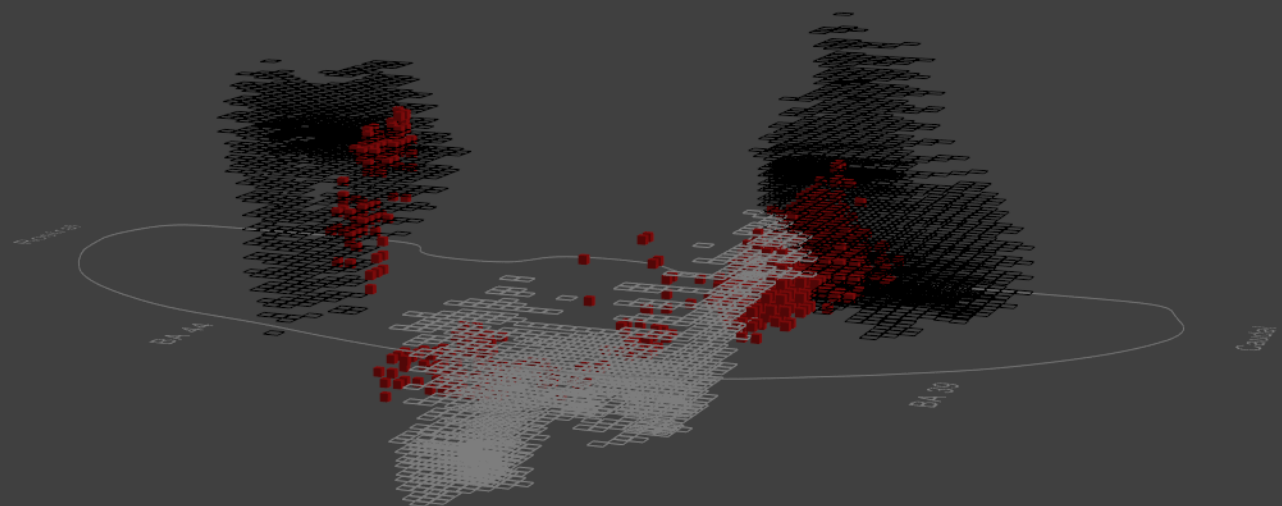

Supplement: Supplementary Data [file supp_awu164_brain-2014-00069-File007.pdf]

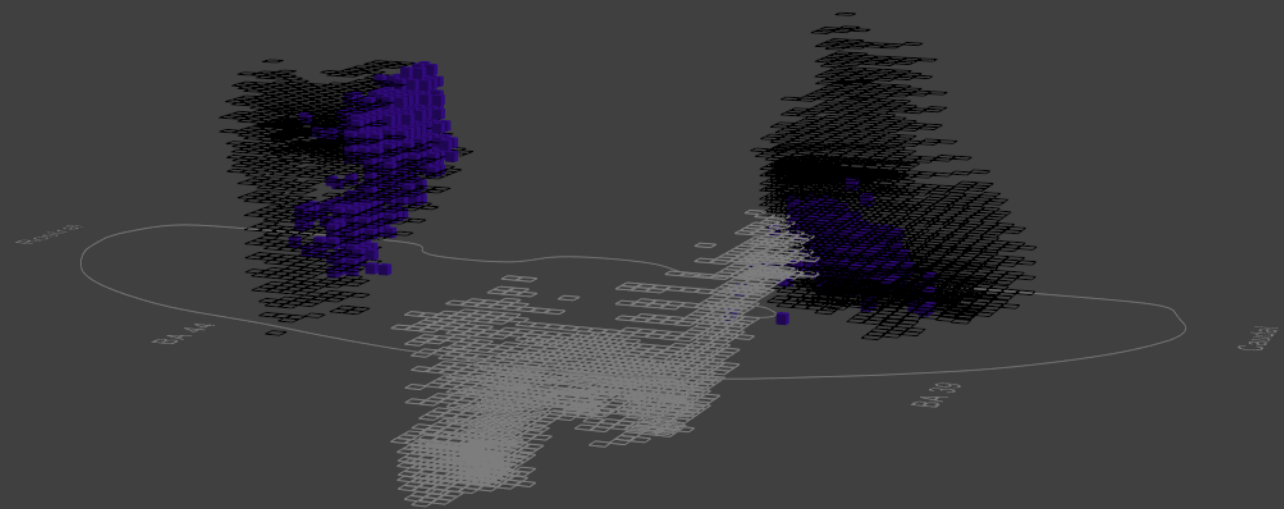

Supplement: Supplementary Data [file supp_awu164_brain-2014-00069-File008.pdf]

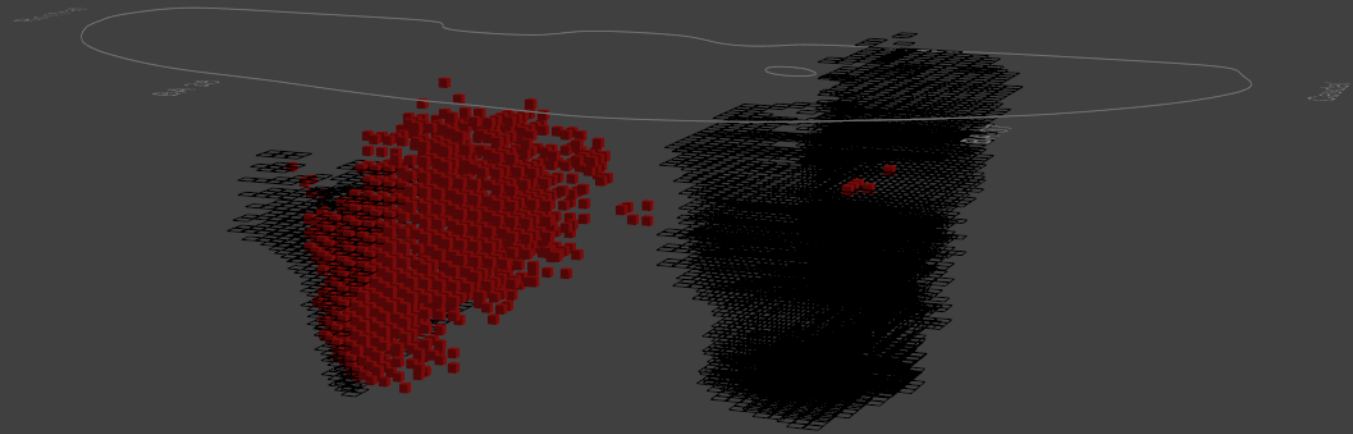

Supplement: Supplementary Data [file supp_awu164_brain-2014-00069-File009.pdf]

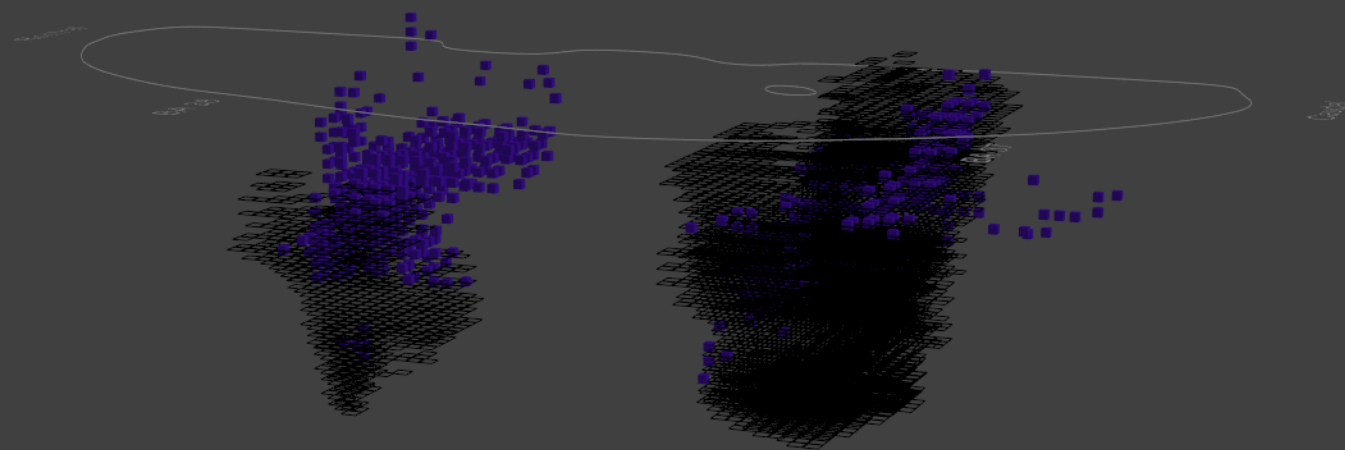

Supplement: Supplementary Data [file supp_awu164_brain-2014-00069-File010.pdf]
